# A Patient Flow Analysis: Identification of Process Inefficiencies and Workflow Metrics at an Ambulatory Endoscopy Unit

**DOI:** 10.1155/2016/2574076

**Published:** 2016-03-29

**Authors:** Rowena Almeida, William G. Paterson, Nancy Craig, Lawrence Hookey

**Affiliations:** Gastrointestinal Diseases Research Unit, Queen's University, Kingston, ON, Canada K7L 2V7

## Abstract

*Background*. The increasing demand for endoscopic procedures coincides with the paradigm shift in health care delivery that emphasizes efficient use of existing resources. However, there is limited literature on the range of endoscopy unit efficiencies.* Methods*. A time and motion analysis of patient flow through the Hotel-Dieu Hospital (Kingston, Ontario) endoscopy unit was followed by qualitative interviews. Procedures were directly observed in three segments: individual endoscopy room use, preprocedure/recovery room, and overall endoscopy unit utilization.* Results*. Data were collected for 137 procedures in the endoscopy room, 139 procedures in the preprocedure room, and 143 procedures for overall room utilization. The mean duration spent in the endoscopy room was 31.47 min for an esophagogastroduodenoscopy, 52.93 min for a colonoscopy, 30.47 min for a flexible sigmoidoscopy, and 66.88 min for a double procedure. The procedure itself accounted for 8.11 min, 34.24 min, 9.02 min, and 39.13 min for the above procedures, respectively. The focused interviews identified the scheduling template as a major area of operational inefficiency.* Conclusions*. Despite reasonable procedure times for all except colonoscopies, the endoscopy room durations exceed the allocated times, reflecting the impact of non-procedure-related factors and the need for a revised scheduling template. Endoscopy units have unique operational characteristics and identification of process inefficiencies can lead to targeted quality improvement initiatives.

## 1. Introduction

The need for screening and surveillance procedures parallels the increase in an aging population and drives the demand for endoscopic procedures in a resource-constrained setting. In 2008/2009, 492,888 gastroscopies, 132,701 sigmoidoscopies, and 969,307 colonoscopies were performed across Canada [1]. The Choosing Wisely Canada campaign, in partnership with the Canadian Association of Gastroenterology Quality Leads, addresses the appropriate use of endoscopic procedures and reflects the paradigm shift toward optimizing value in health care delivery by efficient use of resources [2]. As such, the limitation is paucity of data regarding endoscopic quality and the need for clearly defined, evidence-based processes to facilitate quality improvement in endoscopy consensus reports has been highlighted by the Canadian Association of Gastroenterology, the American Society for Gastrointestinal Endoscopy, and the American College of Gastroenterology [3].

Audits of endoscopy units have revealed significant underutilized resources [4]. However, information is limited and inconsistent on the range of efficiencies of endoscopy units. Zamir and Rex [5] found that specific measures associated with improved procedure volume were reduced room turnover time, use of two rooms per endoscopist, and sedation by a nonendoscopist. This was comparable with the enhanced efficiency observed by Harewood et al. [6, 7] with the combination of postprocedure paperwork elimination, consent and sedation by persons other than the endoscopist, and a two-room model: these changes were shown to translate into increased procedure volume and decreased waiting time. Nevertheless, practice changes that limit the physician-patient interaction portend an undesirable consequence of improved efficiency. Yong et al. [8] assessed the efficiency of the endoscopy unit at Sunnybrook Health Sciences Centre (Toronto, Ontario) over a three-month period and found that, among 675 procedures, delays were a major factor impacting maximal use of endoscopy resources. Up to 31% of their cases were delayed by >15 min; in more than two-thirds of the cases, the delays were physician related. Optimization of factors identified by computer simulation modelling studies, such as room turnover time, patient arrival scheduling, and recovery time, has shown to increase patient volume and reduce patient length of stay [9–11]. More recently, a time-motion study conducted by Day et al. [11] yielded small changes such as reducing appointment times, minimizing recovery room time, and increasing ancillary staff in the preprocedure area that was found to improve efficiency and decrease patient wait times without increasing costs.

Endoscopy efficiency has been challenging due to lack of reliable methodology for efficiency metrics, which is the cornerstone for improving endoscopy value and will facilitate benchmarking best practices [12, 13]. Therefore, to address some of the aforementioned limitations in reliable endoscopy efficiency metrics, a comprehensive analysis of patient flow through the Hotel-Dieu Hospital (HDH, Kingston, Ontario) endoscopy unit was undertaken with a time and motion study. As expected in high-volume procedure units, there are several variables that impact patient flow but are not amenable to quantitative measurement. To bridge this gap in our knowledge and to include nonendoscopist perspectives, focused interviews with individual endoscopy staff were conducted to evaluate their experience of factors that impact efficiency of the unit.

## 2. Methods

### 2.1. Unit Structure and Work Flow

**Figure 1:**
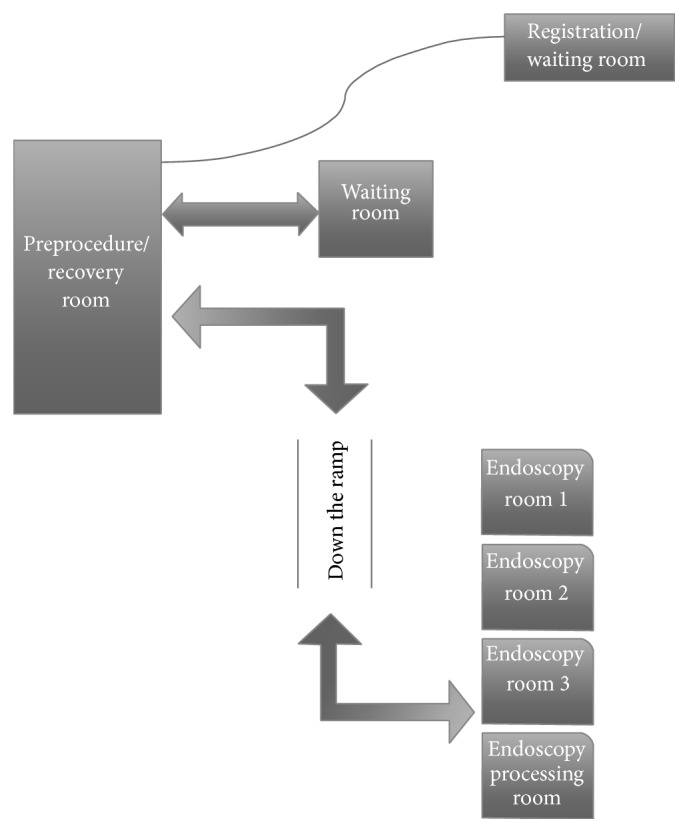
The Hotel-Dieu Hospital (Kingston, Ontario) endoscopy unit workflow. The patient arrives at the registration desk and, on registration, proceeds to the endoscopy suite wherein an endoscopy nurse admits the patient directly either to the preprocedure/recovery room or to the waiting room if a bed is unavailable. Once the preprocedure checklist is completed and the endoscopy room is available, the patient is transported down the ramp to one of the endoscopy rooms.

To develop a process to quantitate and analyze potential inefficiencies within the endoscopy unit, it was first necessary to characterize the different components of the workflow process. As depicted in Figure 1, patients are registered by a receptionist and directed to the endoscopy suite down the hallway. Once at the endoscopy suite, the nurses either admit the patients to the preprocedure/recovery room or assign them to the waiting room if a bed is unavailable. Approximately 80% of the scheduled patients have their assessments completed during a previous clinic appointment, at which time the procedure is explained and written consent has been obtained. The patients who have not been seen in clinic undergo a clinical assessment and informed consent is obtained in the procedure room. An intravenous cannula is placed in the preprocedure room before the patient is transferred to the endoscopy suite. Sedation with midazolam and fentanyl is routinely used and is administered by the nurse in the endoscopy room. After the procedure, the nurse completes paperwork, transports the patient back to the recovery room, and hands the patient over to the nursing staff there. In the endoscopy suite, the endoscopist proceeds with postprocedure paperwork including the procedure report and dictation, while the technicians prepare the room for the subsequent procedure and reprocess the endoscopy equipment in a designated room. Procedure results are communicated to the patient in writing and orally in the recovery room.

### 2.2. Data Collection and Analysis

The present study was undertaken into two distinct stages. During stage 1, a research assistant collected data from December to March 2014 in three distinct settings: the preprocedure/recovery room, endoscopy room, and the overall endoscopy unit room utilization. Procedures were directly observed and the time elapsed for different components from patient registration to exit from recovery room was recorded. The quantitative data were then analyzed for mean times and the 95% CI was calculated.

The second stage included semistructured interviews with the endoscopy unit staff based on standardized interview questions that were drafted for each group (gastroenterology endoscopists, nurses, technicians, and the scheduling assistant). Participants were recruited via email; their interviews were recorded and the transcripts were analyzed for thematic content using NVivo (QSR International, Victoria), a qualitative software program.

The Queen's University Health Sciences Research Ethics Board (Kingston, Ontario) approved the present study and the need for individual patient consent was waived.

### 2.3. Definitions of Endoscopy Workflow Variables

For the purpose of the present study, time intervals representing the endoscopy workflow processes were analyzed; their definitions are outlined in Table 1. In addition to procedure time, intervals for processes pre- and postprocedure were analyzed (e.g., total duration in the preparation room, endoscopy room and the endoscopy unit, preendoscopy time, room turnover, and recovery time). Additional measures of interest for targeted improvement were start time delays such as patient start delay, scheduled start delay, procedure start delay, and endoscopist start delay.

## 3. Results

### 3.1. Unit Demographics

The HDH endoscopy unit is an outpatient facility that operates from 08:00 to 16:00 on weekdays and consists of administrative offices, a waiting room, a 10-bed preprocedure/recovery room, and three endoscopy rooms. It has a closed access scheduling system with block scheduling of procedures and operated as group lists. The preparation/recovery room beds are shared once per week for nonendoscopic procedures such as paracentesis and intravenous infliximab infusions. The daily endoscopy unit staff consists of two to three endoscopists, a variable number of gastroenterology fellows, six nurses, and two technicians. For three days of the week, it is a “two physicians in three rooms” operational model and the other days are staffed with “three physicians in three rooms.” The annual procedure volume of the unit is approximately 5500 cases.

### 3.2. Procedure Measurements

From December to March 2014, 137 procedures in the endoscopy room, 139 procedures in the preprocedure room, and 143 procedures for overall endoscopy unit utilization were collected for 28 nonconsecutive days. All data were analyzed for mean times and 95% CIs (Tables 2 and 3). Approximately 33% of the procedures undertaken had trainee involvement.

The mean duration of time spent by the patient in the endoscopy room before the start of the endoscopic procedure was 14.22 min (95% CI 12.17 min to 16.28 min). The average procedure duration for an esophagogastroduodenoscopy (EGD) was 8.11 min (95% CI 6.64 min to 9.57 min); 34.24 min (95% CI 29.51 min to 38.98 min) for a colonoscopy; 9.02 min (95% CI 7.00 min to 11.05 min) for a flexible sigmoidoscopy; and 39.13 min for a double procedure (95% CI 30.59 min to 47.66 min). The mean overall time spent by the patient in the endoscopy room was 31.47 min (95% CI 26.82 min to 36.12 min) for an EGD; 52.93 min (95% CI 48.11 min to 57.75 min) for a colonoscopy; 30.47 min (95% CI 25.51 min to 35.43 min) for a flexible sigmoidoscopy; and 66.88 min (95% CI 58.64 min to 75.12 min) for a double procedure. The mean room turnover time was 7.9 min (95% CI 7.66 min to 8.14 min). The patient spent an average of 71.21 min (95% CI 63.95 min to 78.48 min) in the preprocedure room and the average recovery room time was 56.27 min (95% CI 53.90 min to 58.64 min). The mean time spent in the endoscopy unit was 2.73 h (95% CI 2.55 h to 2.90 h).

With respect to start time delays, the mean time for patient registration was 39.22 min ahead (95% CI −44.76 to −33.68) of their scheduled start time. The mean scheduled start delay was 19.07 min (95% CI 13.98 min to 24.15 min). The mean procedure start delay was 38.37 min (95% CI 31.34 min to 45.40 min), while the mean endoscopist start delay was found to be 7.51 min (95% CI 5.15 min to 9.86 min). Both the scheduled start delay and procedure start delay were significantly shorter with the two rooms per endoscopist when compared with the one room per endoscopist model (*P* ≤ 0.00).

A subanalysis of the data evaluating start times of the first case of the day shows that the mean duration for the first patient entry into the endoscopy room was 10.49 min (95% CI −2.89 to 23.86) behind scheduled time. Furthermore, the mean start time of the first case of the day was approximately 15 min behind the time of entry of the first patient into the endoscopy room (Table 4).

### 3.3. Structured Interview Results

Focused interviews were conducted with four endoscopists, one nurse sigmoidoscopist, nine nurses, five technicians, and one scheduling assistant. The endoscopy staff work experience varied with the endoscopists ranging from four months to 28 years, four months to 24 years in gastrointestinal endoscopy for the nurses, and five months to 20 years for the technicians.  Table 5 lists the major themes of operational inefficiencies stratified as per endoscopy staff. In their experience, the duration of cases taking longer than the allocated time was identified as the rate-limiting factor that influenced overall patient flow through the endoscopy unit. In the preprocedure room, nursing staff shortage was cited as the major contributory factor. Furthermore, coverage for the nurse and technician during breaks and lunch hours was suggested to alleviate patient flow bottlenecks during these blocks of time. Both nurses and technicians identified the ambiguities of certain tasks, such as the stocking of commonly used supplies in individual rooms, as an overlooked yet frequent source of inefficiency in the endoscopy workflow. The 2 physicians in 3-room model was unequivocally preferred by all groups of staff because it facilitated patient flow, maximized the endoscopist time spent performing procedures, and minimized nonvalue added tasks while waiting for the next patient. Both nurses and technicians identified a strong emphasis on teamwork, communication, and collaboration as the cornerstone for an efficient endoscopy unit.

## 4. Discussion

The present time-motion study observing patient flow through an academic endoscopy unit followed by qualitative interviews yielded baseline endoscopy workflow metrics and process inefficiencies. These data are essential for future resource optimization. We found the cumulative time spent in the endoscopy room was within the allocated time frame for an EGD but far exceeded the allocated times for colonoscopy, flexible sigmoidoscopy, and double procedure. This was despite the procedure times for all except colonoscopies being within reasonable expectations, indicating that non-procedure-related factors are strong determinants of time consumption. This concurs with findings from previous studies that procedure time is rarely rate-limiting and that nonprocedural operational flow processes are instead crucial targets for improvement [11]. Endoscopic procedure duration is unpredictable in highly complex cases and it may be useful to identify variables that are associated with longer procedure times such as sex, age, and previous endoscopic procedure history. Furthering our knowledge of these issues could increase the accuracy of custom scheduling leading to decreased patient waiting times and optimized patient flow.

Recent studies have reinforced that the preprocedure and recovery room are key areas in the endoscopy centre [10, 11]. Given that the average time spent in the preprocedure room was found to be 71 min, reinforcing strategies already in place such as parallel processing of tasks or increasing staff in the preparation room will need to be evaluated [11, 14]. While consent has frequently already been obtained in previous appointments, other approaches, such as sedation by nonendoscopist personnel, are not adopted given that it limits the endoscopist-patient interaction and attenuates the patient-centred model of endoscopic care at HDH. The estimated mean recovery time of 56 min did not account for transportation issues as identified by the staff interviews. For example, at times patients do not arrange for transportation, suggesting the need for reinforced patient education. Limiting recovery time to 30 min did increase procedure volume and provider utilization in the study by Day et al. [11] but was found to be at the expense of increased patient wait time. Hence, there is no consensus on strategies to improve the endoscopy recovery process and it warrants further investigation. Room turnover, often considered to be a critical factor for efficiency, was found to be approximately 8 min in our unit, far less than reported times in the literature [5, 9]. Patient arrival time has been shown to have the most significant effect on patient waiting and resource utilization in discrete event simulation models but was not an issue at our unit [9].

Patient entry into the endoscopy room was approximately 38 min behind scheduled time. This was independent of further delay contributed by endoscopist arrival approximately 8 min later than scheduled start. The start time delays begin with the first case of the day and these incremental delays likely perpetuate a downstream effect on the subsequent scheduled patients. From these data, it was notable that factors such as endoscopist arrival and on-time starts for the first case of the day are modifiable and relatively simple to reinforce to optimize patient flow. The interviews with the endoscopy staff reinforced the observational phase finding that a major shortcoming was related to scheduling and procedure durations exceeding allocated times contributing to downstream patient waiting times. Furthermore, there was objective concordance with staff preference for two rooms per endoscopist given that it was associated with significantly shorter delays.

Operational errors are the product of poorly designed systems as elucidated by the Institute of Medicine's report entitled “To Err is Human: Building a Safer Health System” [15]. Our data unequivocally illustrate the inherent shortcoming of the adopted scheduling system. Predetermined scheduling templates have not evolved to account for case complexity or operator proficiency and lead to misjudgments of daily caseloads. As seen in a simulation model by Day et al. [11], shorter appointment times of 40 min and 35 min are not sustainable without additional resources and unfeasible at 30 min, leading to an infinite patient backlog. While scheduling in the endoscopy unit is a major limiting factor for endoscopy efficiency, there remains insufficient literature in this area. The concept for scheduling for a maximum of 80% utilization of each endoscopy room was forwarded by Larson and Ott [16] and remains in place at most endoscopy units. For example, they scheduled 12 to 14 endoscopy units per room per day whereby an EGD was one unit or an equivalent of 30 min, a colonoscopy was two units, and a polypectomy was assigned colonoscopy plus one unit. Similarly, at the HDH endoscopy unit, the scheduling template is based on the allocation of 15 min for a flexible sigmoidoscopy, 30 min for an EGD or colonoscopy, and 60 min for a double procedure. Scheduling reengineering is a complicated arena, and the utilization of discrete event simulation modelling and queuing theory have yielded principles that merit a trial application to balance patient waiting time with optimization of resources [17].

To address the complexity of variables involved in the endoscopic workflow process, the methodology was specifically designed with a time-motion study to provide a comprehensive assessment of patient flow through the unit. This was supplemented with qualitative information from the entire endoscopy staff to bridge information gaps. This enabled provision of baseline performance metrics and identification of process inefficiencies from multiple perspectives. Some of the limitations of the present study are that it was based at a single ambulatory endoscopy centre and did not directly address some other factors recognized to impact efficiency such as inpatient endoscopic procedures, impact of trainee involvement, or the range of endoscopist proficiencies on the efficiency of the endoscopy unit [5, 18]. Last-minute patient cancellations likely impact efficiency, and prearrival factors are being investigated as a separate study.

Future directions of our research will entail targeted interventions based on the inefficiencies identified and the follow-up effects these have. Correlation with the patient perspective on endoscopy process inefficiencies will also be assessed.

## 5. Conclusion

The present study has identified individual process inefficiencies and endoscopic workflow metrics to facilitate institutional quality control. These baseline data will be essential to move forth from productivity to efficiency and have facilitated the identification of unique opportunities for process improvement at the HDH endoscopy centre.

## Figures and Tables

**Table 1:** Definitions of endoscopy workflow process measures.

Workflow process measures	Definitions
Total duration in preparation room	Time between registration and transfer out of the preparation room

Preendoscopy time	Duration from time of patient entry into endoscopy room until endoscope insertion

Procedure time	Time from endoscope insertion to removal

Total duration in endoscopy room	Duration from actual time of patient entry into endoscopy room until exit

Room turnover time	Time between patient exit and entry of subsequent patient into endoscopy room

Recovery time	Time from patient transfer into the recovery room to exit from the hospital

Total duration in endoscopy unit	Time between registration and exit from the hospital

Patient start delay	Duration between scheduled start time and the actual time of patient registration

Scheduled start delay	Duration between scheduled start time and actual time of entry into the endoscopy room

Endoscopist start delay	Duration between time of patient entry and the time of endoscopist entry

Procedure start delay	Duration between scheduled start time and time of endoscope insertion

**Table 2:** Data regarding endoscopy workflow process measures.

Category	Outcome	*n*	Time, min, mean	95% CI for mean, min
Total duration in preparation room	Duration between registration and time transferred out of preparation/recovery room	116	71.21	63.95–78.48

Preendoscopy time	Duration from patient entry into endoscopy room until endoscope insertion	92	14.22	12.17–16.28

Procedure time (duration from endoscope insertion until removal)	Esophagogastroduodenoscopy	52	8.11	6.64–9.57
	Colonoscopy	45	34.24	29.51–38.98
	Double procedure (esophagogastroduodenoscopy + colonoscopy)	18	39.13	30.59–47.66
	Flexible sigmoidoscopy	11	9.02	7.00–11.05
	Total	126	19.27	16.36–22.17

Total duration in endoscopy room (duration between patient entry and exit from room)	Esophagogastroduodenoscopy	46	31.47	26.82–36.12
	Colonoscopy	52	52.93	48.11–57.75
	Double procedure (esophagogastroduodenoscopy + colonoscopy)	12	66.88	58.64–75.12
	Flexible sigmoidoscopy	19	30.47	25.51–35.43
	Total	129.00	45.44	42.39–48.48

Room turnover time	Duration between patient exit and entry of subsequent patient	93	7.9	7.66–8.14

Recovery time	Duration from patient transfer into recovery room to exit from hospital	98	56.27	53.9–58.64

Total duration in endoscopy unit	Duration between time of registration and time of exit from hospital, h	109	2.73	2.55–2.90

**Table 3:** Data regarding endoscopy workflow start time delays.

Category	Outcome	*n*	Time, min, mean	95% CI
Patient start delay	Duration between scheduled starting time and time of registration	124	−39.22	−44.76 to −33.68
Scheduled start delay	Duration between scheduled start time and time patient transferred into endoscopy room	135	19.07	13.98 to 24.15
Procedure start delay	Duration from scheduled start to endoscope insertion	110	38.37	31.34 to 45.40
Endoscopist start delay	Duration between time of patient entry and endoscopist entry	121	7.51	5.15 to 9.86

**Table 4:** Mean start times of first case of the day.

Outcome	Endoscopy room data, actual time^*∗*^
Scheduled start time of first case of the day	08:00:00
Mean time of first patient entry into endoscopy room	08:02:51
Mean start time for first case of the day	08:17:11
Mean delay for scheduled start of first case of the day	10.49 min (95% CI −2.89 to 23.86)

^*∗*^hh:mm:ss.

**Table 5:** Operational inefficiencies as identified by endoscopy staff.

	Nurses	Endoscopists	Technicians
Most significant change experienced by staff over the years of operation of the endoscopy unit	Increased volume, complexity	Increased volume, complexity	Increased volume, different equipment, dedicated work space

Preferred format	(i) 2 endoscopists to 3 rooms (ii) Full-day endoscopist assignment	(i) 2 endoscopists to 3 rooms	(i) 2 endoscopists to 3 rooms

Preprocedure room/recovery issues	(i) Staff shortage in preparation room (ii) Interruptions from visitors/family (iii) Equipment limited (iv) Limited beds taken up by nonendoscopic procedures (e.g., paracentesis and intravenous infliximab infusions) (v) Transportation		(i) Staff shortage (ii) Preparation room nursing staff variable efficiency (e.g., slower) (iii) Patient arrival time

Endoscopy room issues	(i) Procedures taking longer than scheduled (ii) Total number of cases booked per day (iii) Endoscopist arrival delay (iv) Nursing-related paperwork (v) Running out of supplies (vi) Incomplete endoscopy reporting by endoscopist (vii) Resident teaching (viii) Variable endoscopist efficiency (e.g., speed)	(i) Procedures taking longer than scheduled time (ii) Total number of cases booked per day (iii) Suboptimal staffing (iv) Turnover (waiting for patients from preparation room or to get them to recovery) (v) Resident teaching (vi) Endoscopist arrival delay (vii) Equipment issues (viii) Inconsistent supply stocking (ix) Patient-related delays, cancellations	(i) Procedures taking longer than scheduled time (ii) Total number of cases booked per day (iii) Endoscopist arrival delay (iv) Equipment malfunction (v) Variable endoscopist efficiency (e.g., speed) (vi) Variable endoscopy nurse efficiency

Key attributes	(i) Team work (ii) Communication		(i) Team work (ii) Collaboration

Suggested solutions	(i) Addition of a floating nursing staff for coverage between 11:00 and 14:00 (ii) Break time coverage for the CSR technician (iii) Formalize ambiguous duties, for example, stocking supplies (iv) Porter (v) Move nonendoscopic procedures out of endoscopy space (e.g., paracentesis and intravenous infliximab infusion) (vi) Continuity of endoscopist for full day (vii) Schedule procedures as per endoscopist proficiency	(i) Extra endoscopy room all day (ii) Adequate staffing	(i) Break time coverage for the CSR technician (ii) Formalize ambiguous duties, for example, stocking supplies (iii) Addition of a floating nursing staff for coverage (iv) Scheduling template, for example, avoiding double procedures during lunch hour, too many EGDs in the morning (v) Clarify job description

CSR: central sterile reprocessing; EGD: esophagogastroduodenoscopy.
